# Characterization of Thermal and Electrical Transport in 6.4 nm Au Films on Polyimide Film and Fiber Substrates

**DOI:** 10.1038/s41598-020-66174-8

**Published:** 2020-06-08

**Authors:** Huan Lin, Aijing Kou, Jian Cheng, Hua Dong, Shen Xu, Jingkui Zhang, Siyi Luo

**Affiliations:** 1 School of Environmental and Municipal Engineering, Qingdao University of Technology, Qingdao, Shandong 266033 P.R. China; 2 Automobile Engineering College, Shanghai University of Engineering Science, Shanghai, 201620 P.R. China

**Keywords:** Electronic properties and materials, Electronic properties and materials

## Abstract

The surface and grain boundary scattering impact on the electrical and thermal conduction in the thin metallic films coated on organic substrates has not been studied thoroughly. In this work, we study heat and electron transport in the 6.4 nm thin Au films supported by polyimide (PI) substrate using the transient electro-thermal technique. Thermal and electrical conductivities of 6.4 nm thin Au film are much smaller than bulk value. The thermal and electrical conductivities of 6.4 nm Au film deposited on the PI fiber are reduced by 59.3% and 76.8% in the comparison with the value of bulk Au. For PI film, the reduction of thermal and electrical conductivities is 47.9% and 46.3%. Lorenz numbers of 6.4 nm Au film supported by PI fiber and PI film are 4.51 × 10^−8^ WΩK^−2^ and 2.12 × 10^−8^ WΩK^−2^, respectively. The thermal conductivities of PI fiber and PI film are 0.87 Wm^−1^K^−1^ and 0.44 Wm^−1^K^−1^. The results reveal that PI is a suitable substrate material in the flexible electronic devices field.

## Introduction

With the development of electronic technology, the popularization of intelligent wearable electronic equipment has gradually appeared in our daily life. At present, wearable electronic equipment is widely used in medical detection, sports fitness, communication, entertainment, aerospace and other fields^1^. Conventional electronic devices are generally integrated on a rigid substrate which does not meet the bendable requirements of the wearable electronic equipment^2^. Therefore, developing flexible electronics and improving the performance of flexible electronics equipment are particularly important for the further development of the wearable electronic technology.

In recent years, polyimide (PI) materials have usually been used as substrates in the flexible electronic devices^3^. PI is an organic material with high performance, which possesses a series of great features, such as low thermal conductivity, high tensile strength, tensile modulus^4^, thermal stability^5^, chemical stability, radiation resistance^6^ and insulativity. It is widely used in the fields of high temperature dust removal^7^, marine adiabatic fire protection^8^, aerospace^9^ and machines.

The metallic films have become a research hotspot as an interconnect in the field of semiconductors^10,11^. When the size of grains in the metallic films is approximately equal to the electron mean free path, the interface scattering causes the phenomenon where the electrical and thermal conductivity of the metallic films is much less than the corresponding value of the bulk materials^2,12–18^. Therefore, the Wiedemann-Franz (WF) law which is suitable for bulk materials cannot be used for the thin metallic films^19–21^. Compared with the extensive research on electrical transport^22–24^, there is a lack of research on the in-plane thermal transport of metal films. Au film has excellent thermal and electrical conductivity, and its metal inertia is better than general metal film. Many researchers have studied the properties of Au films. Bediukh *et al*. studied the radio absorbing properties of 10 nm Au film deposited on a dielectric polymer substrate. The Au film showed a high level of absorption, which promotes its application in radar absorbing materials^25^. According to Wang *et al*., Au film is beneficial to further improve performance of sensor^26^. Gu *et al*. reported that 5-nm Au film deposited on the nano-sheet carbon films can improve the field emission characteristics of sample^27^.

In this work, we use the 6.4 nm Au film to study heat and electron transport in the thin metallic films. Firstly, the Au films are coated on the substrate of PI film and fiber by a vacuum sputtering coating apparatus (Q150 TS). Then the transient electro-thermal (TET) technique is used to study electrical and thermal conduction in the Au films supported by PI materials. The results indicate that the thermal and electrical conductivities of 6.4 nm Au film coated on the PI substrate is obviously larger than the value of those deposited on other substrates.

## Materials and Methods

**Figure 1:**
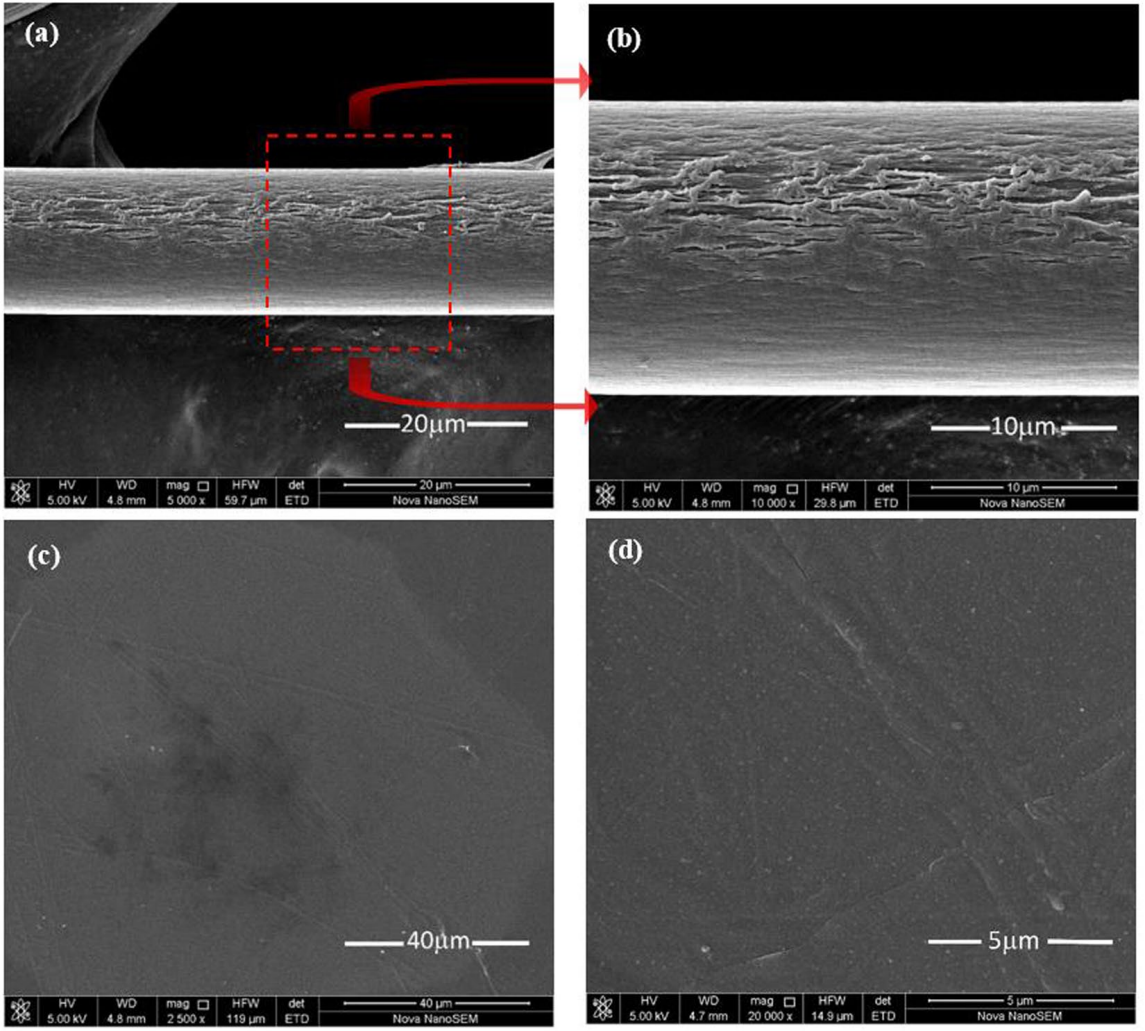
(**a**) The SEM image for the surface texture of PI fiber under low-magnification. **(b)** The high-magnified SEM image of PI fiber. (**c**) The SEM image for the surface texture of PI film under low-magnification. **(d)** The high-magnified SEM image of PI film in this work.

The PI films and fibers used in our experiment are provided by Changchun Hipolyking CO. LTD. The surface texture of the PI fiber and film is determined by using a scanning electron microscope (SEM), as shown in Fig. 1. Through the comparison of the PI fiber and film, we find the surface of PI film is much smoother than PI fiber. The length and diameter of PI fiber are 616.0 μm and 17.1 μm. The length, width and thickness of PI film are 865.5 μm, 58.4 μm and 25 μm, respectively. The specific heat of PI is measured by the differential scanning calorimetry (DSC) as 1090 J (kg•K)^−1^ and the density of PI is 1400 kgm^−3^.

**Figure 2:**
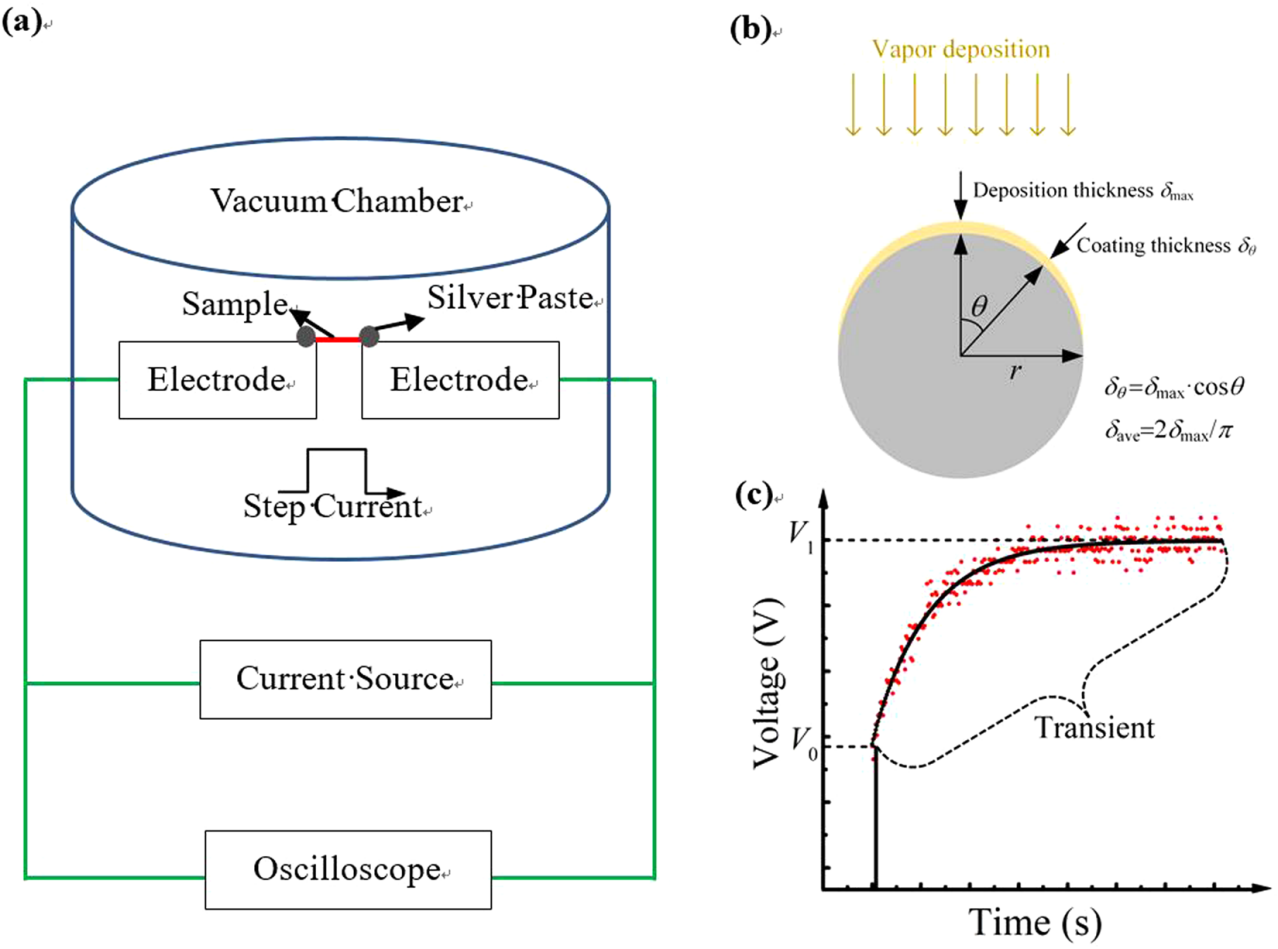
(**a**) The experimental schematic of TET technology in this experiment. (**b**) Profile of the thickness of film deposited on fiber sample. **(c)** The variation of voltage with heating time in the single Au layer. The solid curve and dots are the theoretical fitting and experimental date, respectively.

The TET technique is an effective approach to measuring the thermal diffusivity of one-dimensional materials, containing conductor, semiconductor and nonconductor^28^. For nonconductor, we coat electric film on the top of the sample to make the sample conductive^29^. The Au films are coated on the PI fiber and film by a sputtering coating machine (Quorum 150 T S). The Au atoms are deposited uniformly with a maximum thickness *δ*_*max*_ of Au films coated on the substrate, which is measured during deposition by using a quartz crystal balance. For fibrous substrate, as shown in Fig. 2(b), *δ*_*max*_ of Au films should be on the center top of the fiber, and the average thickness *δ*_*ave*_ of the Au films could be calculated as *δ*_*ave*_ = 2*δ*_*max*_/π^30^. Therefore, *δ*_*ave*_ of Au films is calculated as 6.4 nm, when a *δ*_*max*_= 10 nm Au film is coated on the fibrous substrate. For film substrate, the Au films have an equivalent thickness of *δ*_*ave*_ = *δ*_*max*_. Due to the material is required to be conductive in our measuring technique, the first layer of 6.4 nm Au film was coated on surface of the fiber and film sample. In order to improve measurement and suppress experimental uncertainty, we repeated the process of coating Au film of *δ*_*ave*_ = 6.4 nm four times and obtained four different thermal diffusivities.

The schematic diagram of TET is shown in Fig. 2(a). The sample is suspended between two electrodes and silver paste is used at two connected points to reduce the electrical and thermal contact resistances. The sample is put in a vacuum chamber to reduce the influence of heat convection. During the experimental process, a DC current is fed through the sample to cause the sample heating by using a current source (KEITHEY 6221). The temperature change of the sample results in the evolution of the sample’s resistance, which leads to the variation of the voltage recorded by an oscilloscope (DSO-X3052A). It is well documented that the temperature change over the sample has a close relationship with its thermal diffusivity. As a result, the thermal diffusivity can be determined by using variation of voltage^30^.

The length of PI fiber (616.0 μm) is much larger than its diameter (17.1 μm) and the length of PI film (865.5 μm) is much larger than its width (58.4 μm) and thickness (25 μm). Therefore, the heat transfer in the samples can be simplified as one-dimensional heat conduction along the length direction. So the governing equation for the samples is^31^:

1 $$\frac{1}{\alpha }\frac{\partial \theta (x,t)}{\partial t}=\frac{{\partial }^{2}\theta (x,t)}{\partial {x}^{2}}+\frac{{I}^{2}{R}_{0}}{kLA}+\frac{1}{k}\frac{4U{\varepsilon }_{r}\sigma {\prime} {{T}_{0}}^{3}}{A}\theta ,$$

*α* is the thermal diffusivity of the sample. *T*_0_ is the environmental temperature of vacuum chamber, and *θ* = *T* − *T*_0_. *I* is the current fed through the sample. *R*_0_ is the resistance of the sample before heating. *k* is the thermal conductivity of the sample. *A* and *L* are the cross-sectional area and the length of the sample, respectively. *U* is the circumference of the sample’s cross section. *σ*′ is the Stefan–Boltzmann constant. *ε*_*r*_ is the effective emissivity of the sample.

The voltage variation of the sample is closely related to the average temperature variation of the sample^30^:

2 $${V}_{sample}=I{R}_{0}+I\eta \frac{8{q}_{0}{L}^{2}}{k{\pi }^{4}}\mathop{\sum }\limits_{m=1}^{\infty }\frac{1-{\exp }[-{(2m-1)}^{2}{\pi }^{2}{\alpha }_{{\rm{eff}}}t/{L}^{2}]}{{(2m-1)}^{4}},$$

where *q*_0_ is the electric heating power per unit volume. *η* is the temperature coefficient of the resistance. *α*_eff_ is the measured thermal diffusivity. The normalized overall temperature rise ${T}_{exp}^{\ast }$ of the sample is calculated as ${T}_{exp}^{\ast }=({V}_{{\rm{sample}}}-{V}_{0})/({V}_{1}-{V}_{0})$, where *V*_0_ and *V*_1_ are the initial and final voltage of the sample, respectively. The theoretical normalized temperature rise T^*^ is used to solve the one-dimensional heat transfer problem and it is defined as^32^:

3 $${T}^{\ast }=\frac{96}{{\pi }^{4}}\mathop{\sum }\limits_{m=1}^{\infty }\frac{1-{\exp }[-{(2m-1)}^{2}{\pi }^{2}{\alpha }_{{\rm{eff}}}t/{L}^{2}]}{{(2m-1)}^{4}},$$

when ${T}_{exp}^{\ast }$ is obtained, we can use the different values of *α*_eff_ to fit ${T}_{exp}^{\ast }$ by using Eq. (3). Finally, the value which shows the best fitting of ${T}_{exp}^{\ast }$ is used as the measured thermal diffusivity *α*_eff_ of the sample.

The thermal diffusivity *α*_eff_ obtained in the TET experiment is a combination of the real thermal diffusivity *α* and the value of radiation effect. The real thermal diffusivity can be calculated as^30^:

4 $$\alpha ={\alpha }_{{\rm{eff}}}-\frac{1}{\rho {c}_{p}}\frac{4{\varepsilon }_{r}\sigma {\prime} U{{T}_{0}}^{3}}{A}\frac{{L}^{2}}{{\pi }^{2}}$$

where *ρ* and *c*_*p*_ are the density and specific heat of the sample, respectively.

## Results

The first Au layer coated on the PI fiber is taken as an example to introduce the process of the TET experiment. The length and diameter of the PI fiber are 616 μm and 17.1 μm, respectively. The step current, initial resistance and final resistance of the sample are 0.19 mA, 957 Ω and 968.6 Ω, respectively. The steady temperature of the sample is calculated as 309.6 K. As shown in Fig. 2(c), at the initial process of electrical heating, the value of the voltage changes along with time rapidly and then arrives at a stabilized state. Finally, the thermal equilibrium has been established. The value of measured thermal diffusivity can be determined as 6.27 × 10^−7^ m^2^s^−1^.

**Figure 3:**
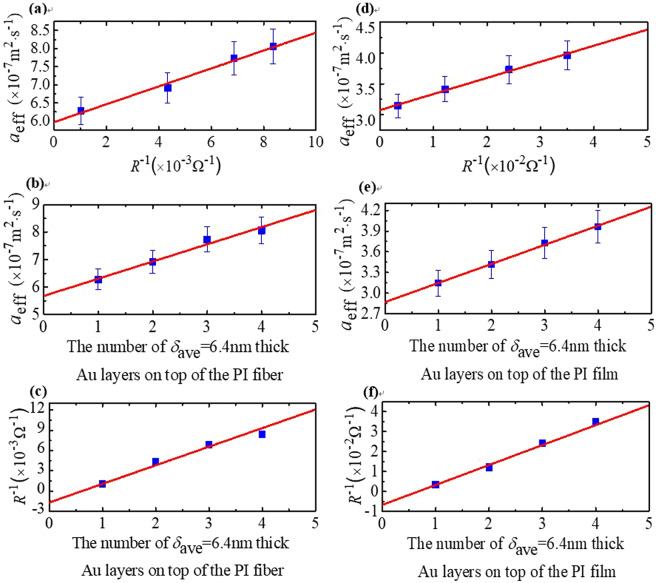
(**a**) The variation of measured thermal diffusivity of 6.4 nm thick Au films coated on PI fiber against the inverse of resistance. **(b)** The linear fitting of the measured thermal diffusivity changes with the number of Au films deposited on the PI fiber. **(c)** The linear fitting of reciprocal of the resistance change with the number of the Au films coated on the PI fiber. In addition, we can obtain the error bars of the Lorenz number, and the electrical and thermal conductivity in our experiment. **(d)** The variation of the measured thermal diffusivity of 6.4 nm thick Au films coated on PI film against the inverse of resistance. **(e)** The linear fitting of the measured thermal diffusivity changes with the number of Au films deposited on the PI film. **(f)** The linear fitting of the reciprocal of the resistance change with the number of the Au films coated on the PI film.

After four times of TET experiments, four sets of *α*, *R* and *n* are obtained. Thermal diffusivity *α* changes with the number of Au films *n* linearly and they have a direct relationship, which can be observed in Fig. 3(b,e) In our experiment, the *σ*_*c*_, *k*_*c*_, *α*_*c*_ and *L*_Lorenz_ of each layer Au films are similar, because they have the same thickness and coated condition.

### Thermal Conductivity of PI Fiber and Film

The measured diffusivity *α*_eff_ is a combination diffusivity of the substrate and Au films^30^:

5 $${\alpha }_{{\rm{eff}}}={\alpha }_{w}+\frac{n\cdot {A}_{c}}{{A}_{w}{\rho }_{w}{({c}_{p})}_{w}}[{k}_{c}-{\alpha }_{w}{\rho }_{c}{({c}_{p})}_{c}],$$

where *α*_*w*_ is the thermal diffusivity of the substrate, *A*_*w*_ and *A*_*c*_ are the cross-sectional area of the substrate and each layer of Au film, respectively. *k*_*c*_ is the thermal conductivity of Au films. *ρ*_*c*_ and (*c*_*p*_)_*c*_ are the density and specific heat of Au films. *ρ*_*w*_ and (*c*_*p*_)_*w*_ are the density and specific heat of PI substrate, respectively. The measured thermal diffusivity *α*_eff_ changes with the number of Au films *n* linearly from Eq. (5) and its y-intercept is *α*_*w*_ as this point has no coating. As shown in Fig. 3(b,e), the intrinsic thermal diffusivities of PI fiber and PI film are 5.68 × 10^−7^ m^2^s^−1^ and 2.87 × 10^−7^ m^2^s^−1^, respectively. Correspondingly, the thermal conductivities of PI fiber and film are calculated as 0.87 Wm^−1^K^−1^ and 0.44 Wm^−1^K^−1^ by using the equation *α*_*w*_ = *k*_*w*_/[*ρ*_*w*_(*c*_*p*_)_*w*_].

### Thermal Conductivity of 6.4 nm Au Films

From Fig. 3(b,e) and Eq. (5), it is obvious that the thermal diffusivity *α*_eff_ linearly varies with the number of Au films *n* and its slope is *A*_*c*_[*k*_*c*_ − *α*_*w*_*ρ*_*c*_(*c*_*p*_)_*c*_]/*A*_*w*_*ρ*_*w*_(*c*_*p*_)_*w*_. The linear fitting slopes of PI fiber and PI film are 6.26 × 10^−8^ m^2^s^−1^ and 2.77 × 10^−8^ m^2^s^−1^, respectively. The density of Au films is 19300 kgm^−3^, and the specific heat is 129 J (kg·K)^−1^. Therefore, the thermal conductivity can be calculated as 129 Wm^−1^K^−1^ and 165 Wm^−1^K^−1^ for the Au films deposited on PI fiber and PI film. The thermal conductivity of bulk Au is 317 Wm^−1^K^−1^. It is found that the thermal conductivities of Au films deposited on PI fiber and film are much smaller than that of bulk Au. This phenomenon is attributed to the electron grain boundary scattering caused by grains in Au films. However, the thermal conductivity of Au films deposited on PI film is closer to the bulk value. Inspired by the references^32,33^, the electron tunneling and hopping in silkworm silk and DNA substrates can enhance the electronic conduction. These substrates are all polarized materials. Therefore, we can get the conclusion that the high thermal conductivity of the Au film deposited on PI films can be explained by the electron hopping and tunneling in the PI film.

### Electrical Conductivity of 6.4 nm Au Films

The electrical resistance of the Au films which is deposited on the substrate can be calculated as:

6 $$R=\frac{L}{n{A}_{c}{\sigma }_{c}}$$

where *A*_*c*_ is the cross-sectional area of each layer of Au film, and *σ*_*c*_ is the electrical conductivity of Au films. Because *A*_*c*_ and *σ*_*c*_ are constant, we obtain the result that *R*^−1^ is linearly against *n*, and its slope is *A*_*c*_*σ*_*c*_/*L*. Figure 3(c,f) also show that the inverse of the resistance changes with the number of Au films linearly^22,30,32,34^. For the Au films on PI fiber and film, the fitted slopes are 2.67 × 10^−3^ Ω^−1^ and 9.97 × 10^−3^ Ω^−1^, respectively. Therefore, we can calculate the electrical conductivity of Au films coated on the PI fiber and PI film as 9.97 × 10^6^ Ω^−1^m^−1^ and 2.31 × 10^7^ Ω^−1^m^−1^.

The electrical conductivities of the Au films coated on PI fiber and film are much smaller than the bulk value 4.3 × 10^7^ Ω^−1^m^−1^. According to the relevant research, the decrease of electrical conductivity is mainly attributed to the grain-boundary scattering^32^. The electrical conductivity of 6.4 nm Au film coated on PI film is 2.3 times that supported by PI fiber.

### Calculation of the Lorenz Number

The thermal diffusivity of sample *α*_eff_ is a combination of Au films and substrate as:

7 $${\alpha }_{{\rm{eff}}}={\alpha }_{w}+{L}_{{\rm{Lorenz}}}TL/[R{A}_{w}{\rho }_{w}{({c}_{p})}_{w}]$$

where *L*_Lorenz_ is the Lorenz number of Au films. It is evident in Eq. (7) that the effective thermal diffusivity (*α*_eff_) changes with the inverse of resistance (*R*^−1^) linearly, and its slope is *L*_Lorenz_*TL*/[*A*_*w*_*ρ*_*w*_(*c*_*p*_)_*w*_]. As shown Fig. 3(a,d), the linear slopes of PI fiber and PI film are 2.47 × 10^−5^ m^2^s^−1^ Ω and 2.60 × 10^−6^ m^2^s^−1^Ω, respectively. Therefore, for Au films coated on the PI film and fiber, the values of *L*_Lorenz_ are 2.12 × 10^−8^ WΩK^−2^ and 4.51 × 10^−8^ WΩK^−2^. The *L*_Lorenz_ of the Au film coated on the PI fiber is much higher than that of bulk Au 2.40 × 10^−8^ WΩK^−2^. We speculate that the grain-boundary scattering has a greater impact on electron transport than on heat transport in the Au-layers coated on the PI fiber.

## Discussion

### Electron Reflection Coefficient for Charge and Thermal Transport

The electrical and thermal conductivity of Au films coated on the glass fiber are 2.71 × 10^6^ Ω^−1^m^−1^ and 61.9 Wm^−1^K^−1^^22^. The *σ*_*c*_ of Au films deposited on the glass fiber has a great diminution of 93.7% from the value of bulk Au 4.3 × 10^7^ Ω^−1^m^−1^ at our experimental temperature^22^. But for the PI fiber and film, the reduction is only 76.8% and 46.3%. The thermal conductivities of the Au films deposited on the glass fiber, PI fiber and film are reduced by 80.5%, 59.3% and 47.9% in comparison with the value of bulk Au. The reduction in electron transport can be interpreted by the Mayadas-Shatzkes (MS) model.

In the MS model^35^, $\frac{{\sigma }_{c}}{{\sigma }_{0}}$ can be expressed as:

8 $$\frac{{\sigma }_{c}}{{\sigma }_{0}}={\left[1+\frac{3(1-p)}{8{k}_{0}}+\frac{7}{5}\alpha \right]}^{-1},$$

The error of this equation is less than 9% for *α* < 10 and *k*_0_ > 0.1, where *α* = *l*_0_*R*′/*d*(1 − *R*′), *k*_0_ = *δ*_*ave*_/*l*_0_, *σ*_*c*_ and *σ*_0_ are the electrical conductivity of Au film and bulk Au. *p* is the specular reflection parameter of electrons at film surfaces, and *R*′ is the electron reflection coefficient at grain boundaries, which means the probability that a conducting electron will be bouncingly reflected when it hits the grain boundary. *l*_0_, *d* and *δ*_ave_ are the electron mean free path of bulk Au, the average grain size, and the average thickness, respectively. The MS model can be used in the film structure consisting of grains which is in a columnar mode to the in-plane direction. This model is feasible for the films deposited by evaporation and sputtering.

**Figure 4:**
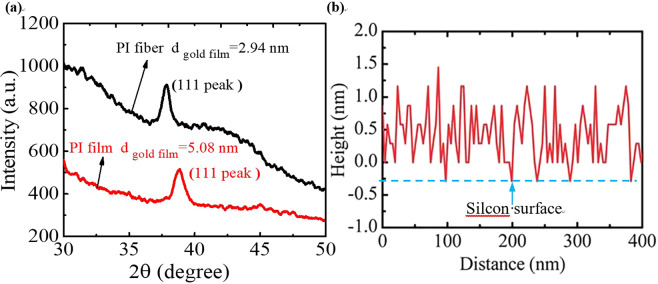
(**a**) The XRD spectrum of the Au film. The black spectrum is the XRD spectrum of the Au films coated on the PI fiber. The red spectrum is the XRD spectrum of the Au films coated on the PI film. **(b)** The surface morphology from AFM of 10 nm Au film coated on the silicon substrate^22^. (Reproduced from ref. ^22^. with permission from The Royal Society of Chemistry).

The average grain size *d* can be calculated by Scherrer formula^36^:

9 $$d=0.89\frac{\lambda }{FW\cdot \,\cos \,\theta },$$

where *FW* is the diffraction peak (111) half height width of the Au film X-ray diffraction (XRD) spectrum in Fig. 4(a). *θ* is the bragg angle, and *λ* is the X-ray wavelength (*λ* = 0.151418 nm).

The electron scattering at Au film surface is specular, and *p* is taken as 1^22,30^. The average thickness of each Au film coated on the PI fiber and film is 6.4 nm. The average grain size *d* of 6.4 nm Au films coated on the PI film and fiber are evaluated as 5.08 nm and 2.94 nm from Fig. 4(a) and Eq. (9). The bulk electrical conductivity *σ*_0_ is 4.3 × 10^7^ Ω^−1^m^−1^ at 308 K^22,37^. The electron mean free path *l*_0_ of bulk Au is calculated as 35.8 nm by using the equation *σ*_0_/*l*_0_ = 1.2 × 10^15^ Ω^−1^*m*^−2^ ^22,38^. Therefore, using the Eq. (8), the electron reflection coefficient at grain boundaries ${R}_{\sigma }^{{\prime} }$ of the Au films coated on the PI fiber and PI film are calculated as 0.16 and 0.08, respectively. Correspondingly, the thermal conductivity of bulk Au *k*_0_ is 317 Wm^−1^K^−1^, and the thermal conductivity is calculated as 129 Wm^−1^K^−1^ and 165 Wm^−1^K^−1^ for the Au films deposited on PI fiber and PI film. The electron reflection coefficient at grain boundaries ${R}_{k}^{{\prime} }$ of the Au films coated on the PI fiber and PI film are fitted as 0.08 and 0.09.

The electron reflection coefficients for charge and thermal transport at grain boundaries of Au films deposited on the PI fiber and PI film are much smaller than on the glass fiber (${R}_{\sigma }^{{\prime} }=0.77$, ${R}_{k}^{{\prime} }=0.48$)^22^, which means the electrons have a higher possibility to pass. It is indicated that electron hopping and tunneling reduce the grain boundary reflection. For PI film, the value of the ${R}_{\sigma }^{{\prime} }$ (0.08) is very close to that of the ${R}_{k}^{{\prime} }$ (0.09). This reveals that the thermal conductivity of the Au films coated on the PI film is mainly dependent on the contribution of electrons. However, for PI fiber, the ${R}_{\sigma }^{{\prime} }$ (0.16) is much bigger than ${R}_{k}^{{\prime} }$ (0.08). The local phonons obtain energy from the electrons reflected by grain boundaries, which improve the contribution of phonon on the total thermal conductivity. Therefore, the influence of electron scattering on thermal conductivity is much smaller than that of electrical conductivity in the Au films coated on the PI fiber.

### The Comparison with Au films on Other Substrates

Table 1 shows the comparison of electrical conductivity, thermal conductivity and Lorenz number between the Au films coated on the PI fiber, PI film, glass fiber, silkworm silk fiber and alginate fiber. It can be observed that the *k*_*c*_ and *σ*_*c*_ of Au films deposited on glass fiber are 61.9 Wm^−1^K^−1^ and 2.71 × 10^6^ Ω^−1^^22^. For the silkworm silk fiber, values of *k*_*c*_ and *σ*_*c*_ are 31.8 Wm^−1^K^−1^ and 4.9 × 10^6^ Ω^−1^, respectively^32^. The *k*_*c*_ and *σ*_*c*_ of Au films coated on the alginate fiber are 75.5 Wm^−1^K^−1^ and 2.63 × 10^6^ Ω^−1^^34^. It is obvious that the *k*_*c*_ and *σ*_*c*_ of Au films deposited on the PI fiber and PI film are much higher than on the glass fiber, silkworm silk fiber and alginate fiber. Especially, the *σ*_*c*_ and *k*_*c*_ of Au films supported by PI film are 8.5 and 2.7 times that supported by glass fiber.

**Table 1:** The thermal conductivity, electrical conductivity and Lorenz number *L*_*Lorenz*_ of 6.4 nm Au films deposited on the substrate of PI fiber, PI film, Glass fiber^a^, Silkworm silk fiber^b^ and Alginate fiber.

Substrate	*δ*_ave_ (nm)	*k*_*c*_ (Wm^−1^K^−1^)	*σ*_*c*_ (×10 ^6^Ω^−1^m ^−1^)	*L*_*Lorenz*_ (×10^−8^WΩK^−2^)
PI fiber	6.4	129	9.97	4.51
PI film	6.4	165	23.1	2.12
Glass fiber	6.4	61.9	2.71	7.44
Silkworm silk fiber	12.8	31.8	4.90	2.08
Alginate fiber	3.2	75.5	2.63	8.66

^**a**^Lin H, Xu S, Li C, Dong H, Wang X (2013) Thermal and electrical conduction in 6.4 nm thin gold films, Nanoscale 5: 4652–4656. 10.1039/c3nr00729d.

^**b**^Lin H, Xu S, Zhang YQ, Wang X (2014) Electron transport and bulk-like behavior of Wiedemann-Franz law for sub-7 nm-thin iridium films on silkworm silk, Acs Appl Mater Interfaces 6: 11341–11347. 10.1021/am501876d.

The electrical conductivity of Au films coated on silkworm silk fiber is approximately twofold the value of Au films coated on glass fiber. But the thermal conductivity of Au films deposited on glass fiber is about twice that coated on silk fiber. It is concluded that the different *k*_*c*_ and *σ*_*c*_ of Au films deposited on the different substrate are not induced by the thickness of films, because the thermal and electrical conductivity of Au films coated on glass and silkworm silk fiber have different patterns of change. This demonstrates that the discrepancies of *k*_*c*_ and *σ*_*c*_ between different substrates are induced by different substrates materials. The thermal and electrical conductivities of Au films coated on the PI fiber and film are much higher than those coated on the glass fiber, silkworm silk fiber and alginate fiber. It is concluded that the electron hopping and tunneling in the PI materials are the main reason for the faster electron transport. In addition, the thermal and electrical conductivities of Au films coated on the PI film are higher than that coated on the PI fiber. The reason is that the smooth surface of the PI film and the uniform thickness of Au films coated on the PI film lead to a better crystallization of Au grains. Through comparison, it is evident that the thermal and electrical conductivities of Au films coated on the PI film are the largest among the five kinds of substrates. So, PI film is an appropriate material to support Au films as a substrate in the field of flexible electronic devices.

The Lorenz numbers *L*_Lorenz_ of the Au films coated on PI fiber, alginate fiber and glass fiber are much higher than that of bulk Au. Therefore, we speculate that grain-boundary scattering has a stronger impact on the electron transport than on the heat transport when we use the PI fiber, alginate fiber and glass fiber as substrates to support Au films. However, in the Au films coated on PI film and silkworm silk fiber, the grain-boundary scattering has the same influence on electron transport and heat transport.

### The Comparison with Thin Au films and Bulk Au

It is obvious that the thermal and electrical conductivities of Au films deposited on the PI fiber and PI film are much lower than the value of bulk Au. We use the theoretical analysis of total electrical resistivity^39^ to explain this phenomenon.

In pure metals, electrons are the main heat carriers. In the case of impure metals or disordered alloys, the contribution of phonons is comparable to that of electrons^40^. The total electrical resistivity (*ρ*_*e*_) is derived from the mechanism analysis of electron scattering, which can be divided into contributions from the isotropic of phonon scattering, grain boundaries, external surfaces, chemical impurities and lattice defects^39^.

10 $${\rho }_{e}={\rho }_{0}+{\rho }_{i},$$

here, *ρ*_0_ is residual electrical resistivity (caused by defect scattering), and *ρ*_*i*_ is intrinsic electrical resistivity (originates from phonons scattering).

The ratio *ρ*_0_/*ρ*_*e*_ can indicate the structural defect degree of the metal sample. The larger the value is, the greater the defect degree of the sample is, which leads to less thermal conductivity. Combined with the SEM images of samples and the above analysis, it can be inferred that Au films deposited on the PI fiber and PI film have a large degree of structural defects, which increases the proportion of electron scattering induced by defects and ultimately leads to a much lower thermal conductivity than the value of bulk Au.

Similarly, the electrical properties of thin Au films are largely reduced by grain boundary scattering, and the defect of Au films deposited on the PI fiber and PI film increases the influence of grain boundary scattering, which results in a much lower electrical conductivity than the value of bulk Au.

### Analysis of Experimental Uncertainty

During the process of coating Au films on the substrate, the thickness of the Au film is monitored by using a quartz crystal microbalance. We coat a thin Au film on a silicon wafer and then measure the thickness by an atomic force microscope (AFM). The Fig. 4(b) shows surface roughness scanning for 10 nm Au films on silicon wafer using AFM. Then, we obtain the same results as the quartz crystal microbalance. The uncertainty of one film’s thickness is less than 10%. In addition, the uncertainty of the measured thermal diffusivity and resistance are less than 6% and 1%^41^. The uncertainties of the measured thermal conductivity, measured electrical conductivity and Lorenz number are less than 12.8%, 6.4% and 11.9%, respectively.

## Conclusions

In our experiment, we have investigated the thermal and electrical conduction in the 6.4 nm Au films deposited on the PI fiber and PI film. The thermal conductivities of Au films coated on the PI fiber and PI film are 129 Wm^−1^K^−1^and 165 Wm^−1^K^−1^, reduced by 59.3% and 47.9% in the comparison with bulk Au. In addition, the electrical conductivities of Au films deposited on the PI fiber and PI film are 9.97 × 10^6^ Ω^−1^m^−1^ and 2.31 × 10^7^ Ω^−1^m^−1^, reduced by 76.8% and 46.3% in the comparison with bulk Au. The thermal and electrical conductivity of Au films coated on the PI film are several times over the values of Au films coated on other substrates. Therefore, PI film has a great potential in the flexible microelectronic device field as a flexible substrate. Moreover, the thermal conductivities of PI fiber and PI film are 0.87 Wm^−1^K^−1^and 0.44 Wm^−1^K^−1^. As a result, this work can also provide guidance for future experimental research.
